# Evaluation of demographic, pathologic, and clinical characteristics and overall survival of patients with colon cancer in Northern Iran (Mazandaran Province) during 2012-2019 

**Published:** 2020

**Authors:** Elahe Rahimi, Jamshid Yazdani Charati, Rezaaali Mohammad pour Tahamtan, Iradj Maleki

**Affiliations:** 1 *Student Research Committee, Faculty of Health, Mazandaran University of Medical Sciences, Sari, Iran*; 2 *Professor of Biostatistics, Health Sciences Research Center, Addiction Institute, Mazandaran University of Medical Sciences, Sari, Iran*; 3 *Asociate professor of Biostatistics, Health Sciences Research Center, Addiction Institute, Mazandaran University of Medical Sciences, Sari, Iran*; 4 *Associate professor ,Gut and Liver Research Center, Faculty of Medicine, Mazandaran University of Medical Sciences, Sari, Iran *

**Keywords:** Colon cancer, Demographic, Clinical, Pathology, Survival analysis

## Abstract

**Aim:**

The present study aimed at evaluating the demographic, pathological and clinical characteristics of patients with colon cancer and also the survival rate and its related factors.

**Background:**

Cancer is the most important barrier to increasing life expectancy in the world. Furthermore, colon cancer is the fourth leading cause of cancer in Iran.

**Methods:**

In this descriptive-analytical study, 219 patients with colon cancer were investigated. Data were analyzed through descriptive and univariate methods using R software. Kaplan-Meier survival analysis and log-rank test were used to evaluate the survival rate of the patients.

**Results:**

In the present study, 25% of patients were below 50 years of age. A family history of cancer was positive in 30% of the patients. According to the clinical symptoms of the patients, rectorrhagia was higher in patients with sigmoid tumor site, abdominal pain was higher in patients with transverse and ascending tumor sites, and anemia was higher in patients with ascending and caecum tumor sites (p< 0.05). The mean life expectancy of the patients was 53.71±2.07 months. Three-year, five-year and seven-year survival rates were 70, 49, and 37 %, respectively.

**Conclusion:**

Half of the patients were diagnosed at advanced stage and the mean survival time at advanced stage was approximately four years. One-third of the patients had local recurrence. It is recommended that patients refer to specialists at specified time intervals for timely diagnosis of the disease and prevention of its recurrence. Providing effective training for people in order for them to acquire more knowledge, and performing screening tests will lead to early diagnosis and lower mortality.

## Introduction

 Non-communicable diseases (NCD) have been the most common cause of human mortality, especially in developed countries, in recent years. Among the non-communicable diseases, cancer is an important factor for reducing life expectancy in all countries, especially in developed and developing countries (1). Colon cancer is the fourth most common cancer and the fifth leading cause of cancer deaths around the world (2). The incidence and mortality of colorectal cancer in Asia is increasing rapidly (3). According to the World Cancer Statistics, in 2018, colorectal cancer is the third most common cancer in Iran, accounting for 9% of all cancers. In the same year, colon cancer in Iran is ranked fourth in terms of incidence and fifth in terms of mortality (4). In low-risk countries such as Iran, the incidence of colorectal cancer has increased over the past three decades. The incidence of colorectal cancer varies depending on the geographic region. There is also a significant difference among different races and ethnic groups in terms of its mortality and incidence (5). The highest incidence of colorectal cancer was found in the central, northern and western provinces of the country and the lowest incidence was seen in southwestern provinces (7, 6). The increasing average age of the population and lifestyle changes are among of the causes of increased colorectal cancer in Iran (8). It is necessary to identify high risk groups and to take effective steps to screen the affected patients and improve the healthcare services provided and prevent this disease, as population, ethnicity and lifestyle vary in different regions of the country and given the high incidence of colorectal cancer in northern Iran. Therefore, the main aim of the present study was to evaluate the demographic, pathological, clinical characteristics and survival rate of colon cancer patients referred to Imam Khomeini Hospital in Sari during the years 2012-2017. These patients had follow up checks until April 2019. 

## Methods

In this descriptive-analytical study, colon cancer patients hospitalized at Imam Khomeini Hospital in Sari during the years 2012-2017 were studied and their colon cancer was diagnosed definitively and follow up checks were carried out until April 2019 over the phone. A checklist was used to collect patient data and the standard of the checklist was confirmed by a medical research consultant. The checklist was completed by examining the patient medical records and via phone calls. The patients who had metastatic colon cancer or benign colon tumors were excluded from the study.

The demographic variables of the research included age at time of diagnosis, sex, place of residence, and history of heart disease, history of diabetes, family history of cancer, history of alcohol use, and history of tobacco use. The clinical, pathologic and therapeutic variables included patients’ clinical symptoms at time of diagnosis, tumor primary site, type of tumor morphology, degree of tumor differentiation, lymph node involvement, and tumor stage, metastasis to other organs, local recurrence, treatment, and resection This information was extracted from medical records of the patients and by asking patients about their health status over the phone. The information was recorded in check lists. Patients had follow-up checks via phone calls and their survival rate was determined as failure time until the end of April 2019. Survival rate was defined as the time between cancer diagnosis until death or last follow-up.

For statistical analysis of the quantitative variables such as age, mean and standard deviation were calculated, and for statistical analysis of the classification variables, such as sex, ratio and percentage were calculated. Chi-square test was used to assess differences in qualitative variables and t-test and ANOVA were used to compare the means in two or more groups. The probability of survival was estimated from diagnosis time until death. Survival analysis in these patients was performed using Kaplan-Meier method and log-rank test. Data were analyzed through R software and the significance level was considered at 0.05. 

## Results

**Table 1:** Demographic characteristics of the patients with colon cancer in northern Iran (Mazandaran province) during 2012-2019

Variable	Sub-groups	Number of patients	Percentage of patients
Age at diagnosis time	17-39	29	13
	40-49	25	12
	50-59	49	22
	60-69	55	25
	70-79	61	28
	Mean ± SD	219	59.3±15.39
Gender	Male	124	57
	Female	95	43
	Male: female ratio	1.3:1	-
Place of residence	urban	141	64
	rural	78	36
History of heart disease	yes	64	29
	no	155	71
History of diabetes disease	yes	49	22
	no	170	78
Family history of cancer	yes	67	31
	no	152	69
History of alcohol use	never	203	93
	Previous or current consumption	16	7
History of tobacco use	never	160	73
	Previous or current consumption	59	27

**Figure 1:**
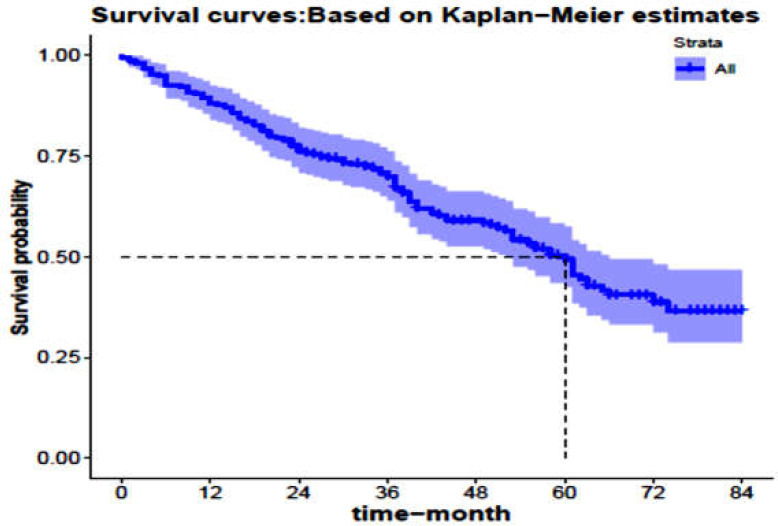
Kaplan-Meier survival curve with 95% confidence interval for the patients with colon cancer in northern Iran (Mazandaran province) during 2012-2019

Out of all patients studied, 25% were younger than 50 years of age, 124 (56%) were male and the sex ratio was 1.3:1. Other data are shown in Table 1. The mean age was 59.89 ± 15.89 years in males and 58.54 ± 14.77 years in females, in other words, no significant difference was seen between males and females (p = 0.51). There was no significant difference between the mean ages in rural areas (59.97 ± 14.43) and urban areas (58.93 ± 15.94) (p = 0.62). The mean age of subjects with a family history of cancer (53.8 ± 16.9) was lower than that in patients without a family history of cancer (61.46 ± 14.11) and this difference was statistically significant (p= 0.001). However, family history of cancer had no significant relationship with sex (p = 0.42). The details of the demographic characteristics are presented in Table 1. 

In terms of the initial symptoms of patients, based on the site of tumor involvement, rectorrhagia was observed more in patients with sigmoid tumor site and this relationship was statistically significant (p <0.001). Abdominal pain was more seen in the patients with transverse and ascending tumor sites and this relationship was statistically significant (p = 0.01). The incidence of anemia was higher in the patients with ascending colon and caecum tumor site and it was statistically significant (p <0.001). Other symptoms had no significant relationship with tumor site.

 According to pathological indicators, cancer morphology in 188 (86%) patients with a mean age of 60.84 ± 14.67 years was adenocarcinoma and mucinous adenocarcinoma was seen in 21 (10%) patients with a mean age of 48.1 ±16.65 years and they had a lower mean age compared to adenocarcinoma and it was statistically significant (p = 0.002). Morphology type had no significant relationship with sex, tumor site and stage of cancer. 

Degree of tumor differentiation was moderate in 125 cases (57%), well in 77 cases (35%) and poor in 17 cases (8%). The rate of well differentiation was seen more in the patients in stage A and poor differentiation was more seen in the patients at stage D and this relationship was statistically significant (p = 0.001). 

**Table 2:** The clinical and pathologic characteristics of the patients with colon cancer in northern Iran (Mazandaran province) during 2012-2019

Variable	Subgroups	n	%
The patient's clinical symptoms at diagnosis time	Rectorrhagia	79	36
	Abdominal pain	63	29
	Change in bowel habits	37	17
	Anemia	20	9
	Weakness and lethargy	7	3
	Weight loss	7	3
	Other cases	6	3
The primary site of the tumor	Caecum	28	13
	Ascending colon	34	15
	Transverse colon	17	8
	Descending colon	27	12
	Sigmoid	85	39
	Involvement of more than one site	28	13
Type of tumor morphology	Adenocarcinoma	188	86
	Mucinous adenocarcinoma	21	10
	Signet ring carcinoma	5	2
	Intra mucosal adenocarcinoma	3	1
	Mucin secreting adenocarcinoma	1	0.5
	Ulcerative adenocarcinoma	1	0.5
Degree of tumor differentiation	well differentiated	77	35
	moderately differentiated	125	57
	poorly differentiated	17	8
Lymph node involvement	N0	127	58
	N1	56	26
	N2	36	16
Tumor stage	A	31	14
	B	78	36
	C	69	31
	D	41	19
Metastasis to other organs	no	146	66
	liver	37	17
	lung	7	3
	Liver and lung	8	4
	Bladder	5	2
	Brain	2	1
	Other	14	7
Local recurrence	yes	72	33
	no	147	67
treatment	Surgery	15	7
	Surgery + Chemotherapy	146	67
	Surgery + Radiotherapy	20	9
	Surgery + Chemotherapy + Radiotherapy	38	17
Resection	partial colectomy	91	42
	total colectomy	45	20
	right hemicolectomy	38	17
	partial resection	31	14
	left hemicolectomy	12	5
	subtotal colectomy	2	1

**Table 3:** The overall survival rate based on cancer stage of the patients with colon cancer in Northern Iran (Mazandaran Province) during 2012-2019

survival rate	1-year	2-year	3-year	4-year	5-year	6-year	7-year
general	0.88	0.76	0.70	0.59	0.49	0.39	0.37
Stage A	0.99	0.87	0.87	0.83	0.83	0.59	0.59
Stage B	0.91	0.83	0.78	0.73	0.67	0.56	0.56
Stage C	0.87	0.77	0.67	0.53	0.44	0.39	0.31
Stage D	0.78	0.54	0.46	0.27	0.06	-	-

**Figure 2:**
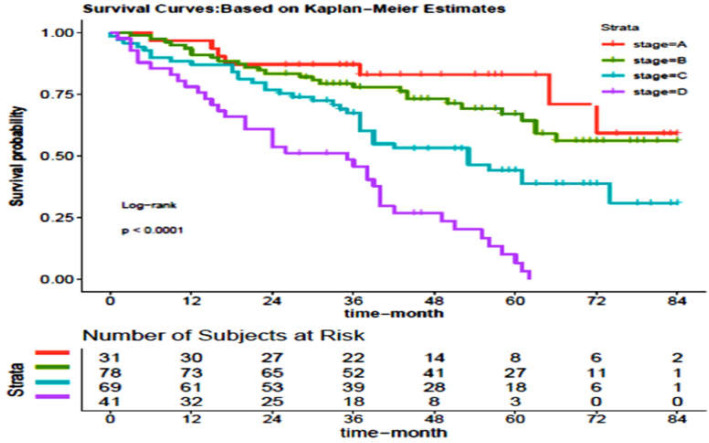
The survival rate of the patients with colon cancer in northern Iran (Mazandaran province) during the years 2012 to 2019 separately in terms of different degrees of cancer at diagnosis time

The degree of tumor differentiation had no statistically significant relationship with sex, age and morphology. 

The number of involved lymph nodes was N0 in 127 cases (58%), N1 in 56 cases (26%) and N2 in 36 cases (16%). There was no significant relationship between the number of lymph nodes involved and sex, age at diagnosis, morphology type and tumor differentiation. 

Most patients (n=78, 36%) were diagnosed at stage B of colon cancer, 69 (31%) patients in stage C, 41 (19%) patients in stage D, and 31 (14%) patients in stage A and cancer stage did not show a significant relationship with sex and age. 

Seventy (34%) patients had tumor metastasis to other organs, of which 36 (51%) had liver metastasis and 19% had metastasis at diagnosis time (15% had metastasis after surgery). At stage D, metastasis was more in liver and lung and it was statistically significant (P <0.001). Seventy-one patients (33%) had local recurrence.

 Sixty-eight percent of patients underwent surgery and chemotherapy, 17% of patients underwent surgery and radiotherapy and chemotherapy, 9% of patients underwent surgery and radiotherapy and 6% of patients underwent surgery alone. The clinical and pathologic characteristics of patients are presented in Table 2.

All subjects had follow-ups. The mean follow-up was 42.10 months and the longest follow-up was 84 months. During the study period, 108 patients died. The mean age of these patients was 60.56 ± 15.48 years, of which 61 (56.48%) were male. Median and mean life expectancy of the patients were 60 and 53.71 ± 2.07 months, respectively. One, three, five, and seven-year survival rate was estimated to be 88, 70, 49, 37%, respectively, as shown in figure 1.

As shown in figure 2, 5-year survival rate was estimated at 83% for stage A, 67% for stage B, 44% for stage C, and 6% for stage D. The details of survival rates are presented in Table 3. 

**Table 4:** Comparison of survival of patients with colon cancer in northern Iran (Mazandaran Province) during the years 2012 to 2019 separately for demographic, clinical and pathologic characteristics

Variable	Sub-groups	Number of patients	Number of died people	Mean survival time ± SE in month	Survival rate			
					36	60	84	P-value
Total	-	219	108	2.07±53.71	0.70	0.49	0.37	-
age during diagnosis	17-49	54	22	4.02±59.1	0.75	0.57	-	0.3
	50-69	104	53	52.8±2.97	0.68	0.52	0.32	
	70-90	61	33	4.04±50.6	0.66	0.38	0.38	
Gender	male	124	61	2.86±53	0.70	0.45	0.42	0.7
	female	95	47	2.97±54	0.70	0.56	0.31	
Place of residence	urban	141	70	2.55±53.5	0.71	0.47	0.34	0.8
	rural	78	38	3.58±54	0.68	0.54	0.40	
History of heart disease	yes	64	30	4.05±53.7	0.69	0.55	0.36	0.8
	no	155	78	2.40±53.7	0.70	0.47	0.36	
History of diabetes	yes	49	20	4.62±57.2	0.69	0.56	0.51	0.3
	no	170	88	2.31±52.8	0.70	0.48	0.33	
Family history of cancer	yes	67	31	3.40±57.7	0.79	0.54	0.38	0.3
	no	152	77	2.57±52	0.66	0.47	0.36	
History of alcohol use	never	203	100	2.08±53.3	0.7	0.50	0.35	0.99
	Previous or current use	16	8	8.28±51.5	0.69	0.50	-	
History of tobacco use	never	160	81	2.40±51.9	0.70	0.45	0.33	0.2
	Previous or current use	59	27	3.95±56.09	0.70	0.58	-	
The patient's clinical symptoms at diagnosis time	Rectorrhagia	79	39	3.30±52.4	0.66	0.48	0.35	<0.001
	Abdominal pain	63	36	3.69±47.4	0.63	0.39	0.27	
	Change in bowel habits	37	19	4.5±53	0.78	0.50	0.33	
	Anemia	20	5	6.17±64.8	0.84	0.77	-	
	Weakness and lethargy	7	1	5.82±74.7	1	0.86	0.86	
	Weight loss	7	2	9.09±66.7	0.86	0.86	0.57	
	Other cases	6	6	6.49±23.8	0.33	-	-	
The primary site of the tumor	Caecum	28	14	5.22±54.7	0.71	0.52	-	0.1
	Ascending colon	34	13	4.97±59.5	0.75	0.62	0.52	
	Transverse colon	17	6	7.25±60.3	0.71	0.71	-	
	Descending colon	27	17	5.65±43.1	0.60	0.25	0.25	
	Sigmoid	85	46	3.21±49	0.68	0.43	-	
	Involvement of more than one site	28	12	5.45±58.3	0.79	0.59	0.39	
Type of tumor morphology	Adenocarcinoma	188	91	1.64±47.4	0.71	0.50	0.39	0.65
	Mucinous adenocarcinoma	21	13	5.21±41	0.57	0.45	-	
	Signet ring carcinoma	5	2	12.26±44	0.6	-	-	
	Other cases	5	2	6.22±54.8	0.75	-	-	
Degree of tumor differentiation	well differentiated	77	33	3.29 ±59	0.74	0.58	0.44	0.006
	moderately differentiated	125	62	2.82±52.9	0.67	0.49	0.37	
	poorly differentiated	17	13	5.23±34.4	0.50	0.25	-	
Lymph node involvement	N0	127	54	2.71±57.8	0.74	0.57	0.44	0.02
	N1	56	30	4.19±51.21	0.67	0.45	0.35	
	N2	36	24	4.53±43.51	0.56	0.23	-	
Tumor stage	A	31	7	4.8±69.3	0.87	0.83	0.59	<0.001
	B	78	27	3.26±63.4	0.78	0.67	0.56	
	C	69	37	.3.69±51	0.67	0.44	0.31	
	D	41	37	3.07±31.1	0.46	0.06	-	
Metastasis to other organs	no	146	45	1.39±49.5	0.85	0.70	0.54	<0.001
	liver	37	31	3.10±34.5	0.50	0.16	0.04	
	lung	7	7	5.96±22	0.14	-	-	
	Liver and lung	8	8	6.18±26.5	0.25	-	-	
	Other organs	21	17	4.42±30.4	0.38	0.08	-	
Local recurrence	yes	72	43	3.23±52.6	0.66	0.45	0.31	0.43
	no	147	65	2.68±54.7	0.72	0.52	0.41	
treatment	Surgery	15	8	8.12±41.3	0.67	0.20	-	0.37
	Surgery Chemotherapy+	146	70	2.39±53.08	0.72	0.50	0.40	
	Surgery Radiotherapy +	20	8	6.74±56.7	0.65	0.59	-	
	Surgery +Chemotherapy +Radiotherapy	38	22	4.34±48.8	0.65	0.47	0.12	
Resection	partial colectomy	91	49	3.15±50.2	0.64	0.43	0.34	0.13
	total colectomy	45	20	4.45±53.6	0.71	0.58	0.22	
	right hemicolectomy	38	14	3.9±64.4	0.84	0.67	0.55	
	partial resection	31	16	5.69±44.4	0.66	0.40	-	
	left hemicolectomy	12	8	8.14±45.1	0.67	0.34	-	
	subtotal colectomy	2	1	14.14±62	1	0.50	-	

A noteworthy relationship was not observed between age at diagnosis time, sex, place of residence, history of heart disease, history of diabetes, family history of cancer, history of alcohol use, history of tobacco use, primary tumor site, type of tumor morphology, local recurrence, treatment, resection and survival rate. However, this rate was affected by the patient's clinical symptoms at diagnosis time, degree of tumor differentiation, lymph node involvement, tumor stage at diagnosis time, and metastasis to other organs (Table 4).

## Discussion

According to the results of the present study, adenocarcinoma tumor was observed in most patients and mucinous adenocarcinoma tumor was observed in 10% of patients who were nearly 49 years old at diagnosis time. In European countries, mucinous adenocarcinoma accounts for 10–20% of colorectal cancer cases and it is most commonly observed in patients under the age of 50 years and in the right colon (9, 10).

The rate of local recurrence in the patients was 33%. Local recurrence leads to death, if left untreated. Its incidence varies depending on the tumor stage (11). Distant metastasis was 15% (except for stage D metastasis) and it was seen more in the liver. In a study conducted by Keshvari et al., the rate of local recurrence and metastasis in colon cancer was reported at 16.44% and 10.96%, respectively (12). A good surgical technique can keep the local recurrence below 10% and keep the metastasis below 20% (13). Since our patients showed a higher percentage of local recurrence and the rate of recurrence depends on several factors, such as the stage of tumor, rate of cell differentiation, and surgical experience, investigating its causes requires a more comprehensive examination.

In the current study, as with other studies, the number of males was higher and the ratio of males to females was 1.3:1 (14, 15, and 16). The mean age of diagnosis was 59 years, 13% of patients were younger than 40 years of age and about 25% of patients were under 50 years of age. In one of the previous studies, the mean age of patients was 52.6 years and 33.4% of patients were younger than 45 (15). In a study conducted by Mirzaeipour et al., the mean age of patients was 58.02 years and 17% of patients were younger than 45 (16), indicating increasing age of disease diagnosis. If it is at a lower stage and higher age at diagnosis time, it will indicate improved lifestyle.

 In the study conducted by Hajmanoochehri et al., the mean age of patients was 57.3 years and 29.2% of the patients were younger than 50 (17). In the study conducted by Fatemi et al., the mean age of patients was 53.5 years (18). In Iran, almost one fifth of all cases of colorectal cancer occur in people younger than 40 years of age (8, 19). The results of the studies in western countries show that 2.6% of colorectal cancer cases occur in people under 40 years of age and 10% in people younger than 50 years of age (20). The results of this study and previous studies in Iran show that the age distribution in Iran is lower than that of western countries. 

 Most patients lived in urban areas, since physical activity was significantly associated with a reduced risk of certain cancers in particular locations, especially colon cancer, and it reduces the risk of the disease by approximately 30-40% (21). People who live in rural areas are more physically active than those living in urban areas and as they have a more vegetable-rich diet, they have reduced risks of cancer.

 A family history of cancer was found in 30% of people and most of them were younger than 53 years of age. In many studies, 24% to 45% of patients had a family history of cancer (14, 15, 16, and 22). 

A positive family history is considered a risk factor for colon cancer (23) and it increases the risk of cancer at ages below 50 (24). In the study conducted by Salimzadeh et al., only 28% of first-degree relatives of patients with colon cancer were aware that they were at risk of cancer and 88% of relatives were not aware about the right age to start screening (25). It indicates poorer knowledge of people about screening. As lack of knowledge and proper training can help increase the incidence of colorectal cancer, the need to increase people’s awareness about early diagnosis for people at risk, especially people with a positive family history, is critical in improving community health. 

In terms of anatomic site, the greatest tumor lesion was in the sigmoid site (38%). In a study conducted by Golfam et al. In 2012, in Tehran, the most common site for colorectal cancer was rectum following sigmoid (26). In Asian countries such as Malaysia and Oman, sigmoid was the most common site involved in colon cancer (27, 28). Rectorrhagia and abdominal pain were the most common clinical symptoms that patients complained of when they referred to medical centers. Initial symptoms of the patients vary depending on the tumor involvement site, so that change in bowel habits is a common symptom in the left colon; whereas, right colon cancer usually shows symptoms such as anemia, iron deficiency, fatigue and weakness. Rectorrhagia often occurs in the rectum and sigmoid. Abdominal pain can occur in tumors grown in all locations (29, 30). This association between patients' initial symptoms and the tumor involvement site is consistent with the results of our study.

About half of the patients referred to medical centers at an advanced stage of their disease (D, C), which can be due to increased risk factors, lack of knowledge of symptoms, ignoring gastrointestinal symptoms, outpatient treatment of gastrointestinal problems, and lack of timely diagnosis and treatment. 

Three-year, five-year, and seven-year survival rates in the patients with colon cancer were estimated to be 0.70, 0.49, and 0.37%, respectively. Akhoond et al. in Tehran reported that 3-year and 5-year survival rates to be 68.5% and 56.8%, respectively (31). Asghari-Jafarabadi et al. in Tehran reported 3-year and 5-year survival rate to be 75.9% and 63.3%, respectively (32). However, in the study conducted by Gohari et al. in Tehran, 3-year and 5-year survival rates were reported to be 71% and 48%, respectively (33). In general, studies in Iran have reported a 5-year survival rate at 48 to 75.2% in the patients with colon cancer (18, 33). In Asian countries such as Malaysia in 2010 and China in 1992-1995, 5-year survival rate in colon cancer was reported at about 44% (34, 35). In a study conducted in Turkey during 2009–2013, 5-year survival rate was reported to be 56.7%. The high survival rate in the mentioned study may be due to the low number of patients (5 patients) at stage D (36). In a population-based study conducted in USA during 2007–2015, the three-year and five-year survival rates were reported to be 70% and 60.02%, respectively (37). One of the reasons for the low survival rate may be the later referral of the patients and the diagnosis of disease at an advanced stage, which leads to lower survival rates. In our study, half of the patients were diagnosed at advanced stages.

 In this study, the five-year survival rate was at 83% for stage A, 67% for stage B, 44% for stage C, and 6% for stage D, which was significant in univariate analysis, so that survival rate decreases with the increase in the stage of disease. In other studies, a strong association has been also reported between tumor stage and survival rate (37-39). In the study conducted by Lee et al. in Taiwan (2007-2007), the five-year survival rate of colorectal cancer patients was 92.2% at stage A, 82.2% at stage B, 63.2% at stage C, and 21.7% at stage D (40). 

It has been found that with increasing lymph node involvement, survival rate decreases. It was also found that the degree of tumor differentiation was effective in survival and the patients with poor differentiation had lower survival rate. Poorly differentiated tumors tend to metastasize, leading to reduced survival in patients; while, well or moderately differentiated tumors have a better prognosis (41). The effect of lymph nodes and the rate of tumor differentiation have been confirmed in various studies (31, 32, 40-42). 

Other studies have also reported that metastasis to other organs had a significant effect on patient survival (41, 42). For example, in the study conducted by Akhoond et al. and Lee et al. (31, 40), metastasis to other organs has been reported as an effective factor. Initial symptoms of the patient at diagnosis time had a significant relationship with survival rate, but the results of this study show no significant relationship between survival rate and the demographic variables included in the study. These results were confirmed in some studies (43, 44); while, differences were reported in some other studies (31, 32, 42). 

Finally, it can be concluded that a significant percentage of patients had a positive family history and about one-third of patients had local recurrence. Further investigations are needed in other studies. Healthy nutrition, exercise, and patient referral at suitable intervals are effective strategies in preventing the disease recurrence. Half of the patients were in stages C and D at diagnosis time. The mean survival time at advanced stages was approximately four years and the survival rate of patients in this province is low, therefore, providing effective training to enhance the knowledge of people with respect to colon cancer and initial symptoms of disease can play a major role in controlling and preventing this type of cancer. Colorectal cancer is a preventable cancer, so it is necessary to identify risk factors and pay attention to gastrointestinal symptoms in patients, do screening tests, especially in people with a positive family history and those who are over 45 years of age. These measures are effective in diagnosing the disease at an early stage, providing better treatment and consequently increasing the survival rate and reducing mortality rate of patients.

## Conflict of interests

The authors declare that they have no conflict of interest.

## References

[1] Bray F, Ferlay J, Soerjomataram I, Siegel RL, Torre LA, Jemal A (2018). Global cancer statistics 2018: GLOBOCAN estimates of incidence and mortality worldwide for 36 cancers in 185 countries. Cancer J Clin.

[2] Rawla P, Sunkara T, Barsouk A (2019). Epidemiology of colorectal cancer: Incidence, mortality, survival, and risk factors. Przegląd Gastroenterol.

[3] Pourhoseingholi MA (2012). Increased burden of colorectal cancer in Asia. World J Gastrointestinal Oncol.

[4] Iran, Islamic Republic of Source: Globocan 2018.

[5] Farhood B, Raei B, Ameri H, Shirvani M, Alizadeh A, Najafi M (2019). A review of incidence and mortality of colorectal, lung, liver, thyroid, and bladder cancers in Iran and compared to other countries. Contemp Oncol.

[6] Shadmani FK, Ayubi E, Khazaei S, Sani M, Hanis SM, Khazaei S (2017). Geographic distribution of the incidence of colorectal cancer in Iran: a population-based study. Epidemiol Health.

[7] Pakzad R, Moudi A, Pournamdar Z, Pakzad I, Mohammadian-Hashejani A, Momenimovahed Z (2016). Spatial Analysis of Colorectal Cancer in Iran. Asian Pacific J Cancer Prev.

[8] Dolatkhah R, Somi MH, Bonyadi MJ, Asvadi Kermani I, Farassati F, Dastgiri S (2015). Colorectal cancer in Iran: molecular epidemiology and screening strategies. J Cancer Epidemiol.

[9] Park SY, Lee HS, Choe G, Chung JH, Kim WH (2006). Clinicopathological characteristics, microsatellite instability, and expression of mucin core proteins and p53 in colorectal mucinous adenocarcinomas in relation to location. Virchows Archiv.

[10] Arai T, Kasahara I, Sawabe M, Kanazawa N, Kuroiwa K, Honma N (2007). Microsatellite‐unstable mucinous colorectal carcinoma occurring in the elderly: comparison with medullary type poorly differentiated adenocarcinoma. Pathol Int.

[11] Papagrigoriadis S (2007). Follow-up of patients with colorectal cancer: The evidence is in favour but we are still in need of a protocol. Int J Surg.

[12] Keshvari A, Fazeli MS, Kazemeini A, Meysamie A, Taromlou N, Kazem M (2014). Evaluation of recurrent colorectal carcinoma after curative resection. Tehran Univ Med J TUMS Publications.

[13] Kraemer M, Wiratkapun S, Seow-Choen F, Ho Y, Eu K, Nyam D (2001). Stratifying risk factors for follow-up. Dis Colon Rectum.

[14] Azadeh S, Moghimi-Dehkordi B, Fatem S, Pourhoseingholi M, Ghiasi S, Zali M (2008). Colorectal cancer in Iran: an epidemiological study. Asian Pacific J Cancer Prev.

[15] Fakheri H, Janbabai G, Bari Z, Eshqi F (2008). The epidemiologic and clinical-pathologic characteristics of colorectal cancers from 1999 to 2007 in Sari, Iran. J Mazandaran Univ Med Sci.

[16] Mirzaeipour A, Salehifar E, Janbabai G, Kouchaki B, Borhani S, Rashidi M (2015). Demographic and clinical characteristics of patients with colorectal cancer. J Mazandaran Univ Med Sci.

[17] Hajmanoochehri F, Asefzadeh S, Kazemifar AM, Ebtehaj M (2014). Clinicopathological features of colon adenocarcinoma in Qazvin, Iran: a 16 year study. Asian Pacific J Cancer Prev.

[18] Fatemi SR, Pourhoseingholi MA, Asadi F, Vahedi M, Pasha S, Alizadeh L (2015). Recurrence and five-year survival in colorectal cancer patients after surgery. Iranian J Cancer Prev.

[19] Malekzadeh R, Bishehsari F, Mahdavinia M, Ansari R (2009). Epidemiology and molecular genetics of colorectal cancer in iran: a review. Arch Iranian Med.

[20] Ansa B, Coughlin S, Alema-Mensah E, Smith S (2018). Evaluation of Colorectal Cancer Incidence Trends in the United States (2000–2014). J Clin Med.

[21] Lee IM (2003). Physical activity and cancer prevention-data from epidemiologic studies. Med Sci Sports Exercise.

[22] Karimi ZA, Saadat A, Jalalian HR, Esmaeili M (2011). Epidemiology and survival analysis of colorectal cancer and its related factors. Kowsar Med J.

[23] Wei EK, Giovannucci E, Wu K, Rosner B, Fuchs CS, Willett WC (2004). Comparison of risk factors for colon and rectal cancer. Int J Cancer.

[24] Slattery ML, Friedman GD, Potter JD, Edwards S, Caan BJ, Samowitz W (1996). A description of age, sex, and site distributions of colon carcinoma in three geographic areas. Interdiscip Int J Am Cancer Society.

[25] Salimzadeh H, Bishehsari F, Delavari A, Barzin G, Amani M, Majidi A (2016). Cancer risk awareness and screening uptake in individuals at higher risk for colon cancer: a cross-sectional study. BMJ Open.

[26] Golfam F, Golfam P, Neghabi Z (2013). Frequency of all types of colorectal tumors in the patients referred to selected hospitals in tehran. Iranian Red Crescent Med J.

[27] Magaji BA, Moy FM, Roslani AC, Law CW (2014). Descriptive epidemiology of colorectal cancer in University Malaya Medical Centre, 2001 to 2010. Asian Pacific J Cancer Prev.

[28] Kumar S, Burney IA, Zahid KF, Souza PCD, Belushi M, Meki TDMWA FM (2015). Colorectal cancer patient characteristics, treatment and survival in Oman- a single center study. Asian Pacific J Cancer Prev.

[29] Saidi H, Karuri D, Nyaim E (2008). Correlation of clinical data, anatomical site and disease stage in colorectal cancer. East African Med J.

[30] Sale A, Saeed MF, Mazin E, Almahmeed EA, Ali AAH, Ali HAH (2018). Unlucky patient combining two rarities: sigmoid colon cancer mimicking appendicular abscess and pseudomembranous colitis involving the small bowel. Int Surg J.

[31] Akhoond MR, Kazemnejad A, Hajizadeh E, Motlagh AG (2010). Comparison of colon and rectum cancer: survival and prognostic factors. Gastroenterol Hepatol Bed Bench.

[32] Asghari-Jafarabadi M, Hajizadeh E, Kazemnejad A, Fatemi S (2009). Site-specific evaluation of prognostic factors on survival in Iranian colorectal cancer patients: a competing risks survival analysis. Asian Pacific J Cancer Prev.

[33] Gohari MR, Biglarian A, Bakhshi E, Pourhoseingholi MA (2011). Use of an artificial neural network to determine prognostic factors in colorectal cancer patients. Asian Pacific J Cancer Prev.

[34] Ghazali AK, Musa KI, Naing NN, Mahmood Z (2010). Prognostic factors in patients with colorectal cancer at Hospital Universiti Sains Malaysia. Asian J Surg.

[35] Xiang Y, Jin F, Gao Y (2011). Cancer survival in Shanghai, China, 1992-1995. IARC Sci Publications.

[36] Kalcan S, Sisik A, Basak F, Hasbahceci M, Kilic A, Kosmaz K (2018). Evaluating factors affecting survival in colon and rectum cancer: A prospective cohort study with 161 patients. J Cancer Res Ther.

[37] Ulanja MB, Rishi M, Beutler BD, Sharma M, Patterson DR, Gullapalli N (2019). Colon Cancer Sidedness, Presentation, and Survival at Different Stages. J Oncol.

[38] Wang Y, Yang L, Zhou M, Shen L, Zhang J, Deng W (2018). Disparities in survival for right-sided vs left-sided colon cancers in young patients: a study based on the Surveillance, Epidemiology, and End Results database (1990–2014). Cancer Management Res.

[39] Roshanaei G, Komijani A, Sadighi A, Faradmal J (2014). Prediction of survival in patients with colorectal cancer referred to the Hamadan MRI center using of Weibull parameter model and determination of its risk factors during 2005-2013. J Arak Univ Med Sci.

[40] Chao-Hsien L, Cheng SC, Hong-Yi T, Chang SC, Ching CY, Shu-Fen W (2018). The Risk Factors Affecting Survival in Colorectal Cancer in Taiwan. Iranian J Public Health.

[41] Nasiri S, Sorush A, Karamnezhad M, Mehrkhani F, Mosafa S, Hedayat A (2010). Prognostic factors in the survival rate of colorectal cancer patients after surgery. Iranian J Surg.

[42] Hassan M, Suan M, Soelar SA, Mohammed NS, Ismail I, Ahmad F (2016). Survival analysis and Prognostic factors for colorectal cancer patients in Malaysia. Asian Pacific J Cancer Prev.

[43] Moghimi DB, Safaei A, Zali MR (2008). Survival rates and prognostic factors in colorectal cancer patients. J Ilam Univ Med Sci.

[44] Li M, Li J, Zhao A, Gu J (2007). Colorectal cancer or colon and rectal cancer?. Oncology.

